# Cardioprotective effects of empagliflozin after ischemia and reperfusion in rats

**DOI:** 10.1038/s41598-021-89149-9

**Published:** 2021-05-05

**Authors:** Jacob Marthinsen Seefeldt, Thomas Ravn Lassen, Marie Vognstoft Hjortbak, Nichlas Riise Jespersen, Frederikke Kvist, Jakob Hansen, Hans Erik Bøtker

**Affiliations:** 1 Department of Cardiology, Aarhus University Hospital, Palle Juul-Jensens Boulevard 99, 8200 Aarhus N, Denmark; 2 Department of Clinical Medicine, Aarhus University, Palle Juul-Jensens Boulevard 82, 8200 Aarhus N, Denmark; 3 Department of Forensic Medicine, Aarhus University Hospital, Palle Juul-Jensens Boulevard 99, 8200 Aarhus N, Denmark; 4 Thunøgade 30, st. tv, 8000 Aarhus C, Denmark

**Keywords:** Heart failure, Myocardial infarction, Experimental models of disease, Cardiology

## Abstract

The Sodium Glucose Co-Transporter-2 inhibitor, empagliflozin (EMPA), reduces mortality and hospitalisation for heart failure following myocardial infarction irrespective of diabetes status. While the findings suggest an inherent cardioprotective capacity, the mechanism remains unknown. We studied infarct size (IS) ex-vivo in isolated hearts exposed to global IR injury and in-vivo in rats subjected to regional myocardial ischemia reperfusion (IR) injury, in whom we followed left ventricular dysfunction for 28 days. We compared rats that were given EMPA orally for 7 days before, EMPA 1.5 h before IR injury and at onset of reperfusion and continued orally during the follow-up period. We used echocardiography, high resolution respirometry, microdialysis and plasma levels of β-hydroxybutyrate to assess myocardial performance, mitochondrial respiration and intermediary metabolism, respectively. Pretreatment with EMPA for 7 days reduced IS in-vivo (65 ± 7% vs. 46 ± 8%, p < 0.0001 while administration 1.5 h before IR, at onset of reperfusion or ex-vivo did not. EMPA alleviated LV dysfunction irrespective of the reduction in IS. EMPA improved mitochondrial respiration and modulated myocardial interstitial metabolism while the concentration of β-hydroxybutyric acid was only transiently increased without any association with IS reduction. EMPA reduces infarct size and yields cardioprotection in non-diabetic rats with ischemic LV dysfunction by an indirect, delayed intrinsic mechanism that also improves systolic function beyond infarct size reduction. The mechanism involves enhanced mitochondrial respiratory capacity and modulated myocardial metabolism but not hyperketonemia.

## Introduction

Although mortality from acute MI has declined over the last 25 years^1,2^, long-term mortality, i.e. 1 year and beyond, and post-MI heart failure is still significant^1,3^. The disarrays that may lead to deterioration of left ventricular (LV) contractile function are determined by the extent of myocardial damage caused by MI^4^. The underlying mechanisms are multifactorial and involve not only complex remodeling at the organ level but also neurohormonal changes to compensate for the lack of contractile function^5^.

Recent groundbreaking clinical studies have shown that treatment with Sodium Glucose Co-Transporter 2 (SGLT2) inhibitors markedly reduced cardiovascular mortality and hospitalization in patients with HF^6–9^. The reduction was independent of diabetic status^8,9^. While originally intended as a glucose-lowering alternative to metformin for patients with type II diabetes mellitus, contemporary discoveries indicate that SGLT2 inhibitors have a class effect on HF in individuals surviving an acute MI, whereas the incidence of recurrent MI is unchanged. The mechanisms underlying these observations are unknown.

Supporting the clinical findings, a recent experimental study demonstrated that the SGLT2 inhibitor, canagliflozin, has a cardioprotective capability by reducing infarct size in an in vivo rat model of ischemia reperfusion (IR) independently not only of circulating glucose but also circulating ketone levels^10^ that are known to be increased by SGLT2 inhibitor treatment^11^. Despite absent infarct size reduction in an in vitro rat heart model, another SGLT2 inhibitor, empagliflozin (EMPA), improves post-MI mitochondrial respiration and increases inner membrane permeability in non-diabetic rats^12^. The ischemic myocardium is characterized by suppressed mitochondrial oxidative phosphorylation, which may be further deranged by reperfusion^13^. We hypothesized that cardioprotection by SGLT2 inhibitors is associated with improved mitochondrial respiration. Consequently, the aim of the present study was to investigate the effect of EMPA on IS and cardiac and mitochondrial function after IR induced chronic ventricular dysfunction and after acute IR injury in an in vivo rat heart model.

## Materials and methods

### Animals and design

Male Sprague Dawley rats (250–300 g, Taconic, Ry, Denmark) were kept for acclimatization at a constant temperature of 23 °C, with a 12 h light–dark cycle and with unlimited access to food and water.

Our study included three experimental series:

1. Impact of EMPA (30 mg/kg) on infarct size in vivo and ex vivo was investigated to determine if the cardioprotection by EMPA, if any, was dependent on a whole-body system or could be induced directly on the heart. We compared rats receiving chronic oral EMPA treatment daily for 7 days before MI (EMPA-Chronic) to rats receiving an acute oral administration 1.5 h before MI (EMPA-Acute) or at the onset of reperfusion (EMPA-Post). Placebo animals were matched in dose and time of administration of vehicle (Supplementary Fig. S1a).
2. Impact of EMPA on post-MI LV dysfunction after 28 days evaluated by echocardiography and high resolution respirometry for mitochondrial respiratory capacity. In this series we compared EMPA-Chronic, EMPA-Post, a sham group receiving EMPA (SHAM) and corresponding placebo animals. In all groups in this experimental series, the administration of EMPA (30 mg/kg) continued consecutively for 28 days after myocardial infarction (Supplementary Fig. S1b).
3. Impact of EMPA on mitochondrial respiratory capacity measured by high resolution respirometry and myocardial intermediary metabolism measured by microdialysis during early reperfusion (30 min) was investigated to delineate the metabolic changes caused by EMPA treatment. In this series we compared EMPA-Chronic, a matched placebo group (PLACEBO-Chronic), EMPA-Acute and a sham group (SHAM) (Supplementary Fig. S1c).

Times of administration were chosen for several reasons. To test the most acute effect of EMPA we chose to administer EMPA 1.5 h before intervention, since the drug reaches maximum bioavailability after 1 h^14^. To test any effect requiring longer time and repeated administrations we chose to administer EMPA repeatedly for 7 days before intervention. Also, levels of β-OHB were measured at the different time points to determine the relation to any potential cardioprotection.

Exclusion criteria included death during procedure, death during follow up, complications to oral gavage, no sign of myocardial infarction evaluated during procedure; no paling or hypokinesia, no sign of living mitochondria during high resolution respirometry, increase of > 10% in the oxygen consumption rate after addition of cytochrome C during high resolution respirometry, coronary flow rate > 20 ml/min in the isolated perfused heart model indicating unspecific leakage, incorrect microdialysis probe placement and leaking probe membrane.

### Experimental models

#### In vivo rat model

Rats were anaesthetized in an induction chamber with 8% sevoflurane (Sevorane, AbbVIE A/S, Copenhagen, Denmark) mixed with atmospheric air (flow: 0.8 L/min). Upon induction of anaesthesia, the rats were intubated and connected to a mechanical ventilator (Ugo Basile 7025 rodent ventilator, Comerio, Varese, Italy) with an adjusted flow of 0.6 L/min with 3.5% sevoflurane. A temperature probe (UNO, Zevenaar, Holland) was inserted, and body temperature was kept at a constant 37 °C ± 1 °C. Prior to the procedure the rats were injected subcutaneously with buprenorfin (Temgesic, Reckitt Benckiser Pharmaceuticals Limited, Slough, England) (0.045 mg/mL) to ensure peri-, and post-operational analgesia. A left sided thoracotomy, via the fourth and fifth rib, followed by pericardiotomy gave access to the myocardium. The left anterior descending coronary artery (LAD) was located and ligated approximately 2 mm distally to the junction between the pulmonary conus and the left atrial appendage using a 4-0 silk suture (Sofsilk, Covidien, Dublin, Ireland)^14^. Myocardial paling and hypokinesia distally to the ligature confirmed ceased blood flow and ischemia. After 30 min of ischemia, reperfusion of the myocardium was ensured by removing the ligature and confirmed by visualization of hyperaemia and enlargement of the LAD. In animals used for infarct size evaluation, the ligature was only loosened but remained in the chest during reperfusion, to ensure exact delineation of the area at risk (AAR). After reperfusion the chest was closed. Animals in the long-term survival group were given buprenorfin (7.4 μg/ml) in the drinking water for three days following MI.

#### Preparation of the isolated perfused heart

The hearts were isolated and perfused ex vivo as previously described^15^. In brief, rats were anaesthetised by a subcutaneous injection of Dormicum (midazolam (0.5 mg kg^−1^ body weight); Matrix Pharmaceuticals, Herlev, Denmark) mixed with Hypnorm (fentanyl citrate (0.158 mg kg^−1^ body weight) and fluanisone (0.5 mg Kg^−1^ body weight)). The rats were tracheotomized and connected to a mechanical ventilator and ventilated with atmospheric air (Ugo Basile 7025 rodent ventilator, Comerio, Varese, Italy). A thoracotomy and laparotomy were performed and a bolus of 500 IU heparin (Leo Pharma, Ballerup, Denmark) was administered through the femoral vein. Subsequently, the ascending aorta was cannulated and retrogradely perfused at a constant pressure of 80 mmHg with an oxygenated (95% O_2_ and 5% CO_2_) Krebs Henseleit buffer: composition in mM: NaCl 118.5, KCl 4.7, NaHCO_3_ 25.0, glucosemonohydrate 11.0, MgSO_4_⋅7 H_2_O 1.2, CaCl_2_ 2.4 and KH_2_PO_4_ 1.2. The heart was excised and transferred under continuous perfusion to an isolated perfused heart system (IH-SR type844/1; HSE, March-Huhstettem, Germany) with a constant temperature of 37 °C. An intraventricular balloon (size 7, HSE, March-Hugstetten, Germany) was inserted in the LV through the left atrial appendage and the volume adjusted to a LV end-diastolic pressure of 4–8 mmHg to simulate preload. Data was digitally converted (DT9804; Data Translation. Marlboro, MA, USA) and stored using Notocord Hem software (version 2.0, Notocord systems, Croissy sur Seine, France).

#### Infarct size

In the in vivo model, the ligature around LAD was tightened again after 2 h of reperfusion, and a 4% Evans Blue (Sigma-Aldrich, St. Louis, MO, USA) solution was injected in vena cava inferior, to outline the myocardial area at risk (AAR), as previously described^15^.

The hearts were excised, frozen at − 80 °C and sliced into 5, 2 mm thick slices, guided by a rat heart slicer matrix (Zivic Instruments, Pittsburgh, PA, USA). The slices were then incubated for 3 min with 1% 2,3,5-triphenyltetrazoliumchloride (TTC) (Sigma-Aldrich, St. Louis, MO, USA) to delineate areas of infarction and after 24 h of storage in 4% formaldehyde buffer (VWR International, Leuven, Belgium), AAR and IS were assessed using ImageJ software (NIH, Bethesda, Maryland, USA).

In the isolated perfused heart model, the same protocol was followed without Evans Blue staining, since the hearts were subjected to global no flow ischemia. IS/AAR, AAR/LV and IS/LV were calculated and adjusted to the wet weight of the individual slices.

#### Echocardiography

Transthoracic echocardiography was performed at baseline, day 1 and day 27 after MI, as previously described^15^. Transthoracic echocardiography was performed with a Vevo 2100 high-frequency ultrasound system (Visual Sonic, Toronto, ON, Canada) with a 21 MHz rat probe. Animals were lightly sedated (3% sevoflurane, atmospheric air), fixated in a supine position to a heating pad and connected to ECG electrodes and a rectal thermal probe. Movement of the transducer was facilitated by a mechanical setup to eliminate movement disturbances. Two-dimensional and M-mode images were obtained.

Left ventricular volumes in end diastole (LV Vol_d_) and end systole (LV Vol_s_) were calculated using the bullet method (5/6 × LV Vol_d_/LV Vol_s_ area x LV length). Left ventricular ejection fraction (EF) was calculated using LV Vol_d_ and LV Vol_s_ by the formula:

$$EF(\mathrm{\%})=\frac{\left(LV Vo{l}_{d}-LV Vo{l}_{s}\right)}{LV Vo{l}_{d}}*100$$

All images were analyzed using the Vevo 2100 software.

#### Mitochondrial respiratory capacity

We analyzed the mitochondrial respiratory capacity with non-fatty acid and fatty acid substrates using high-resolution respirometry (Oxygrah-2 k; Oroboros Instruments, Innsbruck, Austria) as described previously^16^. A mid-papillary biopsy of LV myocardial tissue was prepared by manual dissection of fiber bundles (~ 1.5 m), 30 min after MI in series 3 and 28 days after MI in series 2. In series 3 fibers were dissected at mid papillary level central in the area at risk, to ensure evaluation of mitochondria exposed to IR. In series 2 fibers were dissected from the remote myocardium, since fibrotic areas are not suited for analysis.

Fiber bundles were permeabilized in cold BIOPS solution mixed with saponin (50 μg mL^−1^) by gentle agitation for 30 min^16^. After permeabilization the fiber bundles were rinsed twice for 10 min by agitation in a cold respiration medium, MiR05 (in mmol L^−1^: 110 sucrose, 60 K-lactobionate, 0.5 EGTA, 0,1% BSA, 3 mgCl_2_, 20 taurine, 10 KH_2_PO_4_ and 20 Hepes; pH 7.1).

The fibre bundles were added to the chambers in the Oxygraph-2 k respirometer, filled with 2 mL MiR05. Substrates and inhibitors were added in the following order for non-fatty acid linked oxidation: (1) Glutamate (G) (10 mmol L^−1^) + Malate (M) (2 mmol L^−1^), (2) ADP (5 mmol L^−1^), (3) Cytochrome c (10 μmol L^−1^), (4) Succinate (S) (10 mmol L^−1^), (5) Oligomycin (complex V inhibitor) (2 μg mL^−1^), (6) Rotenone (complex I inhibitor) (0.5 μmol L^−1^) + Antimycin A (complex III inhibitor) (2.5 mmol L^−1^).

We also measured the capacity of mitochondrial fatty acid oxidation using substrates in the following order: (1) Octanoyl-L-carnitine (250 μmol L^−1^), a medium chain fatty acid, + Malate (2 mmol L^−1^), (2) ADP (5 mmol L^−1^), Cytochrome c (10 μmol L^−1^).

To avoid any O_2_ limitations to respiration the chambers were hyperoxygenated and all measurements were carried out in duplicate. The integrity of the outer mitochondrial membrane was tested by adding cytochrome c and an increase of > 10% in the oxygen consumption rate led to exclusion. The respiratory rates (O_2_ consumption rates) are expressed as the O_2_ flux normalised to the cardiac muscle mass of the permeabilized fibers (pmol s^−1^ kg^−1^ wet weight of permeabilized fibers).

GM: complex I respiration without ADP. GM3: Complex I respiration with ADP. GMS3: Complex I + II respiration with ADP (maximal coupled respiration). 4o: LEAK/non-phosphorylating resting respiration. ROX: residual oxygen consumption. Moc: fatty acid respiration without ADP. Moc3: fatty acid respiration with ADP. The respiratory control ratio (RCR) was calculated as state 3 respiration/state 2 respiration and expresses the respiratory coupling efficiency of the electron transport system (ETS) independent of muscle weight and mitochondrial density.

#### Mitochondrial enzymatic activity

We analyzed citrate synthase (CS) activity in the cardiac tissue by spectrophotometry as described previously^16^. The results are expressed as μmol min^−1^ g protein^−1^.

#### Ketone body assay

β-hydroxybutyrate (β-OHB) was quantified in rat plasma using hydrophilic interaction liquid chromatography tandem mass spectrometry as previously described^17^. The blood sample was taken from the left femoral vein immediately prior to excision of the heart.

#### Myocardial interstitial concentrations of metabolites assessed by microdialysis in vivo

Myocardial interstitial concentrations of metabolites were assessed by microdialysis as described previously^18^. After placement of the ligature around the LAD in the in vivo model but before inducing ischemia, a microdialysis probe (membrane length 4 mm, cut-off 6 Da; AgnTho’s, Lidingoe, Sweden) was carefully guided by a 26 g needle into the free anterior wall of the LV in the estimated area of infarction. The probe was connected to a microdialysis pump (Univentor Limited, Zejtun, Malta) and perfused at a flow speed of 1μL min^-1^ with deoxygenated KHB (95%N_2_ and 5% CO_2_). Following insertion, a 20-min period of constant flow was allowed for stabilization, to ensure the concentrations of myocardial metabolites reach equilibrium. We measured concentrations of the TCA cycle intermediates; citrate, malate and succinate and ATP degradation products; hypoxanthine, xanthine, adenosine and inosine, during stabilization, ischemia and reperfusion at 10-min intervals and stored samples at − 80 °C. During collection the vials were cooled to approximately 4 °C. Samples were analysed by liquid chromatography and mass spectrometry. The absolute values were corrected to recovery rate as described previously^19^.

#### Myocyte cross sectional area

Hematoxylin/eosin (HE) staining was used for visualization of myocyte cross-sectional area.

Briefly, cross sections of the left ventricle (3 µm) were deparaffinized and counterstained with hematoxylin II (Ventana Medical Systems, AZ, USA) 28 days after MI.

Myocyte cross-sectional area was evaluated at 400 × magnification in the HE stained sections. Cells were measured manually by outlining the cell contour. Only cells with a visible nucleus, a clear and intact cell membrane and cells located perpendicular to the plane were measured. Five to seven randomly selected fields in each section were selected for evaluation in the remote myocardium, which was characterized as the area most distally from the infarcted area.

Microscopy was performed on a light microscope (BX50F4, Olympus, Tokyo, Japan) and image analysis was performed using Image J software (NIH, Bethesda, MD, USA).

#### Drug preparation and administration

EMPA (30 mg/kg) (Empagliflozin, Merck, Darmstadt, Germany)^20^ was dissolved in a 0.5% hydroxyethylcellulose (Sigma-Aldrich, St. Louis, MO, USA) solution and administered by oral gavage. Placebo animals received vehicle only. The relatively high dose of EMPA was based on pivotal non-clinical safety studies^20^, a pilot trial in (Supplementary Fig. S2) and since this study serves as a proof-of-concept study exploring the mechanism of action rather than the clinical potential of EMPA.

### Statistical analysis

Based on own experience and reports by other groups, a sample size of n = 10 was considered adequate to identify a treatment effect^12,15,21^. All results are expressed as mean ± SD unless otherwise stated. Comparisons of means between three or more groups were analysed by one-way ANOVA with post-hoc Bonferroni test. Comparison between two groups was done using a student’s t-test. Concentrations of myocardial interstitial metabolites were analysed using two-way ANOVA. All analyses were performed using GraphPad Prism 8.2.0 (Graph Pad Software, CA, USA). P < 0.05 was considered statistically significant.

### Ethics approval

All animal handling was in accordance with national guidelines in Denmark and the Guide for the Care and Use of Laboratory Animals^37^ and all experiments conformed to Danish Law (Act. No. 1306 of 23/11/2007) and the experimental setup was approved by The Animal Experiments Inspectorate, Ministry of Environment and Food of Denmark with the license number: 2018-15-0201-01475. All animal experiments were carried out in accordance with the ARRIVE guidelines.

## Results

In the in vivo infarct size study, we included 66 rats. Numbers for the final analysis and those excluded are shown in the flow chart in supplementary figure S1a. In the ex vivo infarct size series, we included 20 animals. Two rats were excluded due to protocol violations. In the post MI LV-dysfunction series, we included 50 rats. The flow chart is shown in supplementary figure S1b. In the mitochondrial respiratory capacity and intermediary metabolism series, we included 50 rats. Supplementary Figure S1c depicts the flow chart and specifies reasons for exclusion of 21 rats.

**Figure 1:**
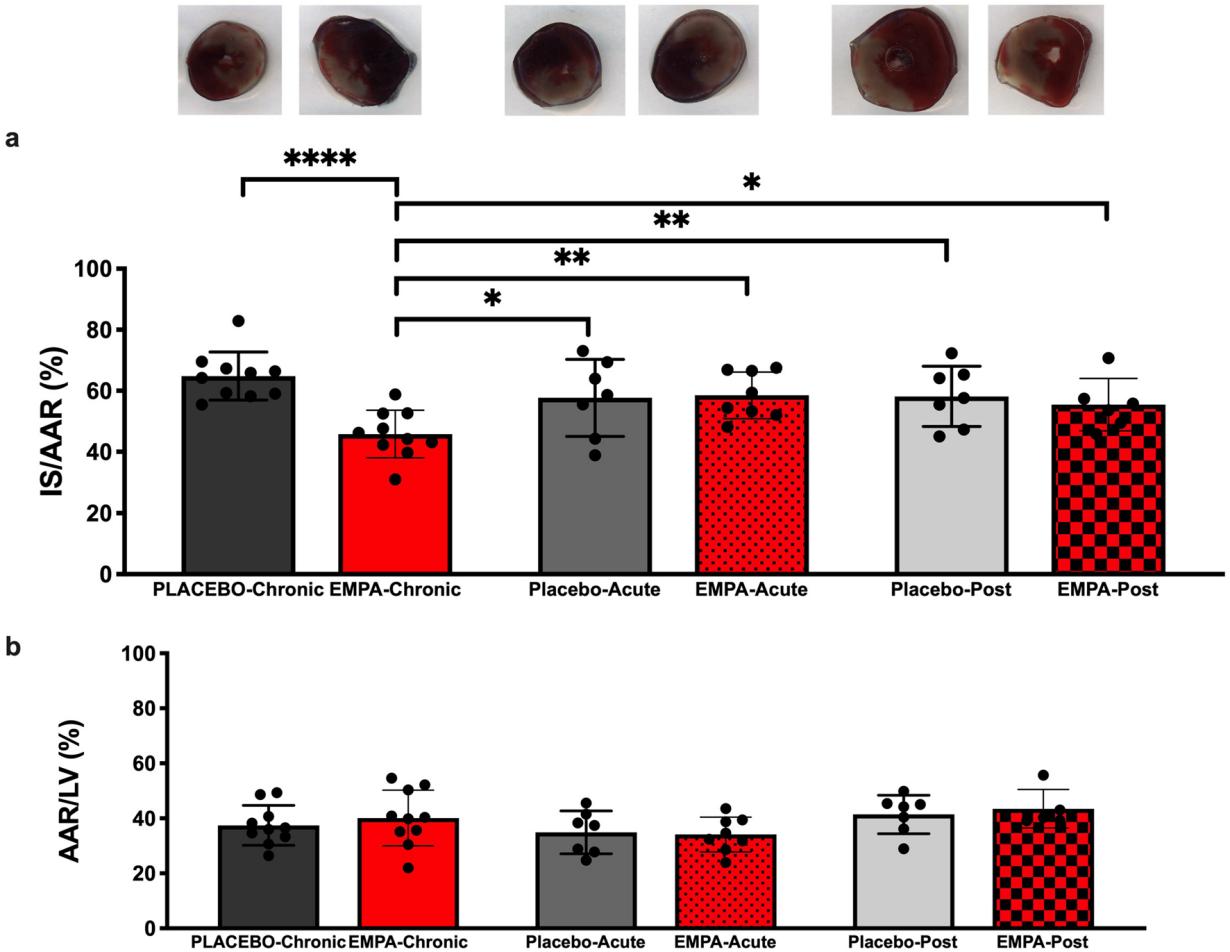
Myocardial infarct size following ischemia reperfusion in vivo. (**a**) Histological evaluation of infarct size with 2,3,5-triphenyltetrazolium chloride (TTC) staining, 2 h after reperfusion in PLACEBO-Chronic (n = 10); EMPA-Chronic (n = 10), PLACEBO-Acute (n = 7), EMPA-Acute (n = 8), PLACEBO-Post (n = 7) and EMPA-Post (n = 6) (**b**) The ratio of AAR/LV indicating the similarity of AAR between groups. *AAR* area at risk, *IS* infarct size, *LV* left ventricle. Mean ± SD. Statistical significance is shown as * p < 0.05, ** p < 0.01, **** p < 0.0001.

EMPA treatment for 7 days prior to MI significantly reduced myocardial in vivo IS by 20% points compared to placebo (65 ± 7% vs. 46 ± 8%p < 0.0001) (Fig. 1a). Administration of EMPA 1.5 h before MI yielded no IS reduction in vivo (p > 0.99) (Fig. 1a). AAR did not differ between groups (Fig. 1b). EMPA did not reduce IS ex vivo (63 ± 16% and 53 ± 13%, p = 0.14) or affect hemodynamic performance (Supplementary Fig. S3).

**Figure 2:**
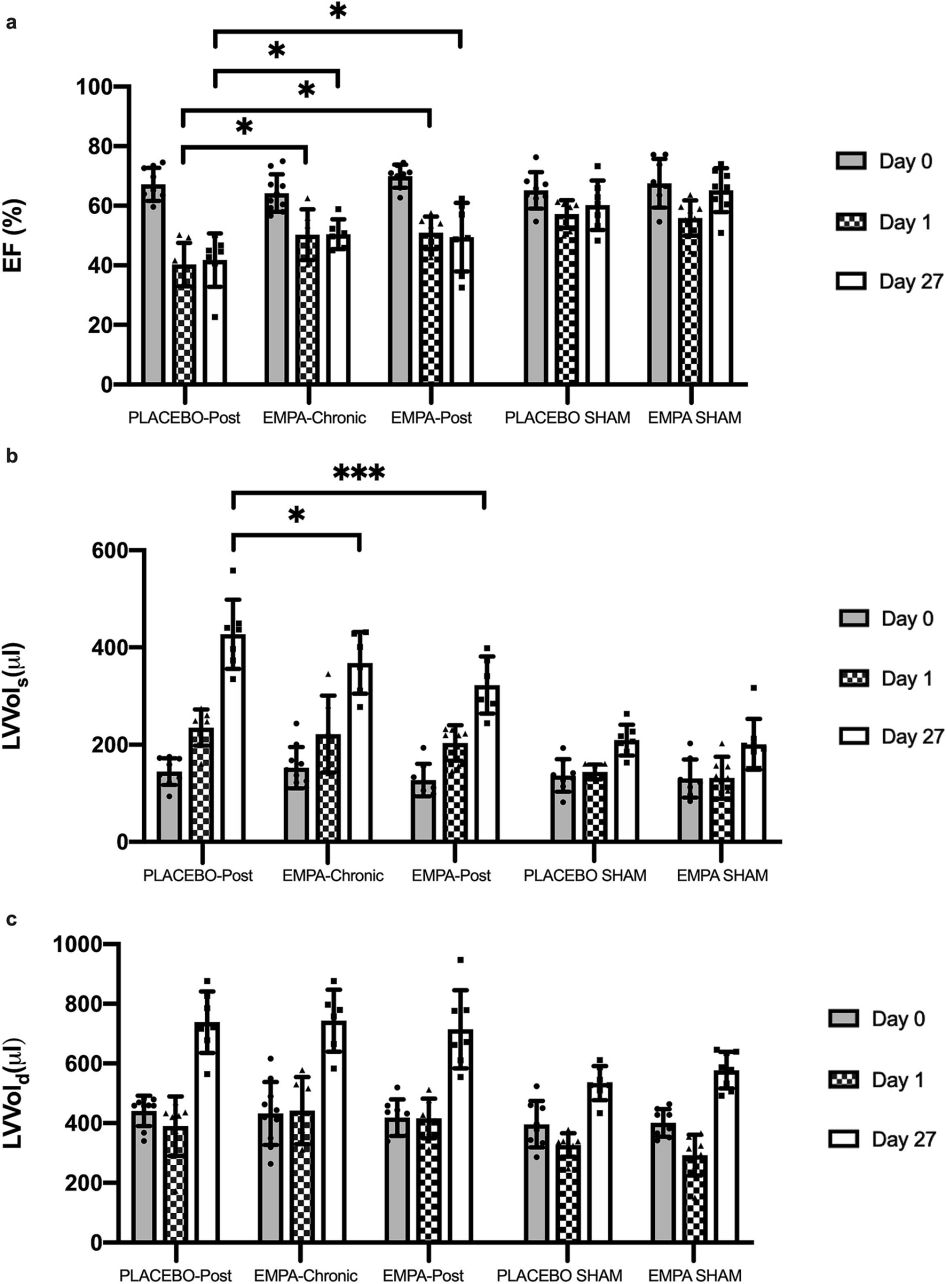
Effects of EMPA on left ventricular function. Effects of EMPA on (**a**) left ventricular ejection fraction, left ventricular volumes in (**b**) end systole and (**c**) end diastole were assessed by echocardiography in PLACEBO-Post (n = 7), EMPA-Chronic (n = 6), EMPA-Post (n = 8), PLACEBO SHAM (n = 7) and EMPA SHAM (n = 7). *EF* ejection fraction, *LV Vol*_*s*_ left ventricular volume in the systole, *LV Vol*_*d*_ left ventricular volume in the diastole. Mean ± SD. Statistical significance is shown as * p < 0.05, *** p < 0.001.

**Figure 3:**
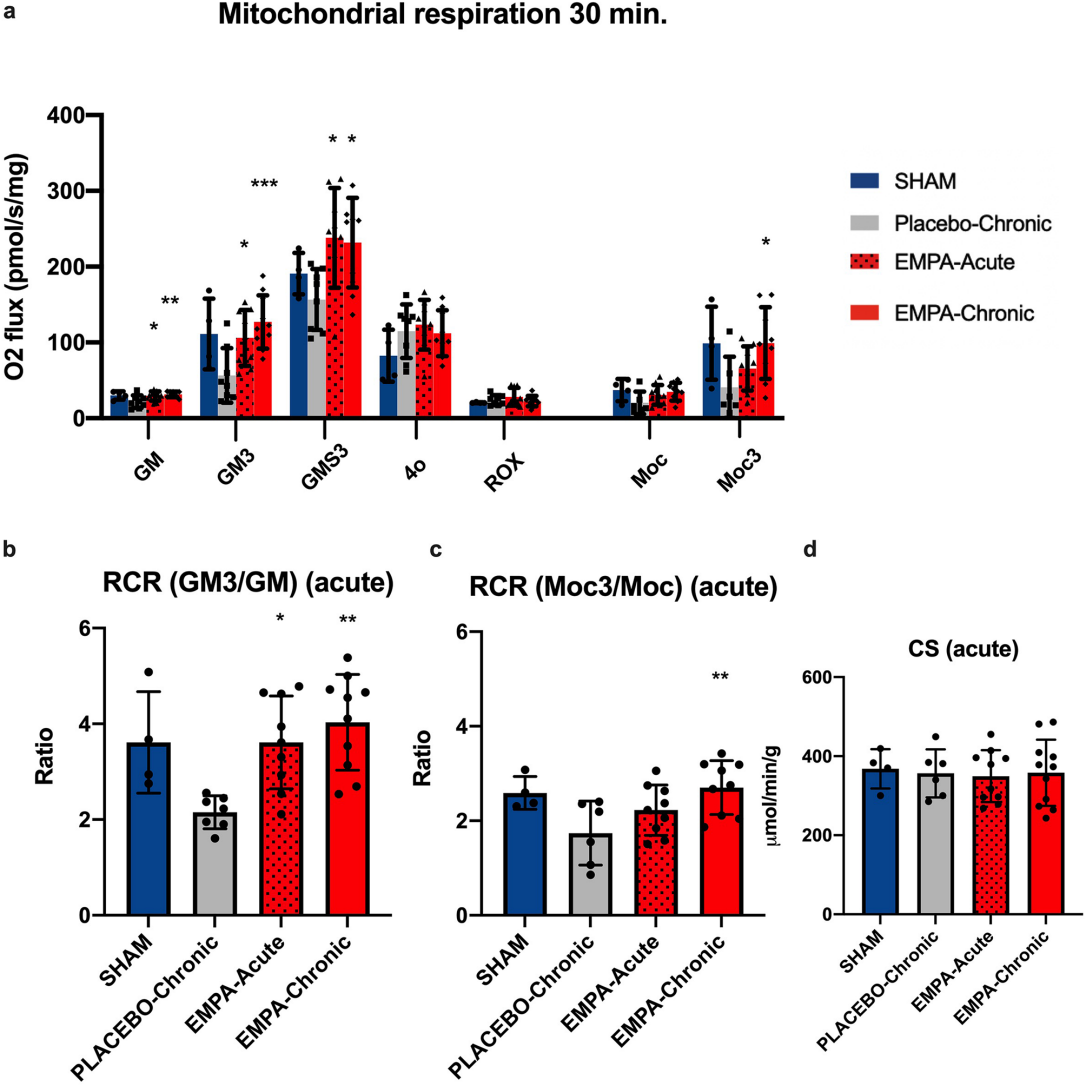
Mitochondrial respiratory capacity in early reperfusion. (**a**) Summarized data of mitochondrial respiration in each respiratory state. (**b**) RCR with complex I linked substrates. (**c**) RCR with complex I + II linked substrates. (**d**) Enzymatic activity of CS. SHAM (n = 4), PLACEBO-Chronic (n = 8), EMPA-Acute (n = 9), EMPA-Chronic (n = 10). *GM* Complex I respiration with glutamate and malate and ADP, *GM3* Complex I respiration with glutamate, malate and ADP, *GMS3* Complex I + II respirations with glutamate, malate, succinate and ADP, *4o* LEAK/non-phosphorylating respirations with oligomycin, *ROX* residual oxygen consumption evaluated after adding rotenone and antimycin A, *Moc* fatty acid respiration with malate and octanoyl-L-carnitine without ADP, *Moc3* fatty acid respiration with malate, octanyol-L-carnitine and ADP. RCR respiratory control ratio, CS citrate synthase. Data are mean ± SD. Statistical significance is shown as * p < 0.05, ** p < 0.01, *** p < 0.001 vs. PLACEBO-Chronic.

LV EF was similar in all groups at baseline. LV EF was reduced after MI at day 1 and day 27 compared to baseline in the placebo (p < 0.0001), EMPA-Chronic (p = 0.0005) and EMPA-Post (p < 0.0001) groups (Fig. 3a), whereas there was no reduction in the sham groups between baseline and day 27 (p = 0.34). EMPA (EMPA-Chronic and EMPA-Post) significantly improved LV EF at day 1 and at 27 after MI compared to placebo (50 ± 5 and 49 ± 11 vs. 41 ± 9%, p < 0.05) (Fig. 2a).

Neither LV Vol_s_ nor LV Vol_d_ differed between groups at baseline (Fig. 2b,c). Animals treated with EMPA (EMPA-Chronic and EMPA-Post) had significantly improved LV Vol_s_ at day 27 after MI compared to placebo (368 ± 63 and 322 ± 59 vs. 427 ± 71 $\mu L$, p < 0.05) (Fig. 2b). We observed no difference in LV Vol_d_ between the EMPA treated and placebo groups at day 27 after MI (Fig. 2c).

Compared to placebo EMPA-Chronic and EMPA-Acute increased non-ADP stimulated complex I respiration (31.6 ± 3.7 and 29.4 ± 6.3 vs. 21.7 ± 8.3 mol$\rho$ O_2_ s^−1^ mg^−1^, p < 0.05) (Fig. 3a) as well as ADP stimulated complex I respiration (127.1 ± 35,16 and 106.1 ± 36,86 vs. 56.4 ± 36,01 mol$\rho$ O_2_ s^−1^ mg^−1^, p < 0.05) with glucose linked substrates 30 min after MI. Accordingly, the RCR increased (4.0 ± 0.99 and 3.6 ± 0.97 vs. 2.2 ± 0.34, p < 0.05) (Fig. 3b).

Stimulation of complex I + II yielded an enhanced response in both EMPA-Chronic and EMPA-Acute compared to placebo (231.7 ± 59.04 and 238.1 ± 65.77 vs. 156.6 ± 40.25 mol$\rho$ O_2_ s^-1^ mg^-1^, p < 0.05). State 4o respiration (non-phosphorylating respiration) and residual oxygen consumption (ROX) were similar between all groups.

Respiration with fatty acid substrates 30 min after MI was similar between all groups in complex I (non-ADP stimulated) linked respiration (Fig. 3a). In contrast, EMPA-Chronic increased complex I + II (ADP stimulated) respiration compared to placebo (99.2 ± 47.3 vs 40.8 ± 40.3 mol$\rho$ O_2_ s^−1^ mg^−1^, p < 0.05) (Fig. 3a). This was reflected in an increased RCR by EMPA-Chronic (2.7 ± 0.6 vs. 1.7 ± 0.7, p < 0.05) (Fig. 3c).

**Figure 4:**
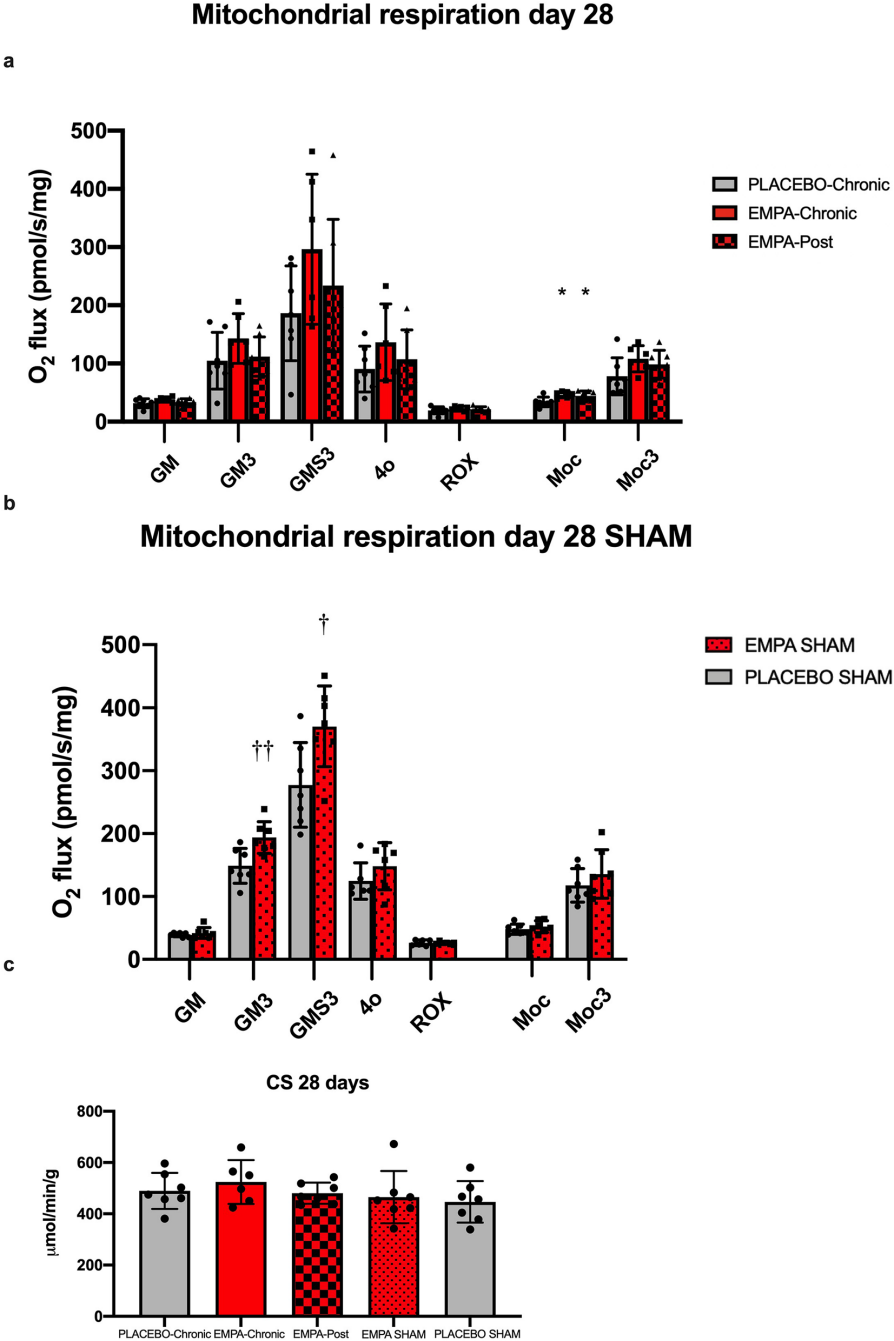
Mitochondrial respiratory capacity at day 28 after reperfusion. (**a**) Summarized data of mitochondrial respiration in each respiratory state. (**b**) Mitochondrial respiratory capacity at day 28 after sham operation. (**c**) Enzymatic activity of CS. PLACEBO-Chronic (n = 7), EMPA-Chronic (n = 6), EMPA-Post (n = 7), EMPA SHAM (n = 7), PLACEBO SHAM (n = 7). *GM* State 2 respirations with glutamate and malate, *GM3* State 3 respirations with glutamate and malate, *GMS3* State 3 respirations with glutamate, malate, and succinate, *4o* State 4 respirations with oligomycin, *ROX* residual oxygen consumption evaluated after adding rotenone and antimycin A, *Moc* state 2 respiration with malate and octanoyl-L-carnitine, *Moc3* state 3 respiration with malate and octanyol-L-carnitine, *RCR* respiratory control ratio, *CS* citrate synthase. Data are mean ± SD. Statistical significance is shown as * p < 0.05, ** p < 0.01, *** p < 0.001 vs. PLACEBO-Chronic. † p < 0.05, †† p < 0.01 vs. PLACEBO SHAM.

At 28 days after MI EMPA-Chronic increased ADP stimulated complex I + II respiration with glucose linked substrates, but the increment was not statistically significantly different from placebo (296.3 ± 128.9 vs. 183.9 ± 81.$\rho$ mol O_2_ s^−1^ mg^−1^, p = 0.17), (Fig. 4a).

Respiration with fatty acid linked substrates demonstrated an increase of complex I respiration (non-ADP stimulated) by EMPA-Chronic and EMPA-Post compared to placebo (46.6 ± 5.4 and 44.5 ± 8.3 vs. 33.2 ± 8.2 mol$\rho$ O_2_ s^−1^ mg^−1^, p < 0.05) (Fig. 4a).

SHAM animals were evaluated after 28 days of exposure to either EMPA or vehicle.

Complex I (ADP stimulated) respiration (193.8 ± 25.2 vs. 148.8 ± 27.8 mol$\rho$ O_2_ s^−1^ mg^−1^, p = 0.008) as well as complex I + II (ADP stimulated) respiration (370.3 ± 64.0 vs. 277.4 ± 67.2 mol$\rho$ O_2_ s^−1^ mg^−1^, p = 0.02) were increased by EMPA compared to placebo (Fig. 4b).

Enzymatic activities of CS did not differ between either of the experimental series (Figs. 3d, 4c).

**Figure 5:**
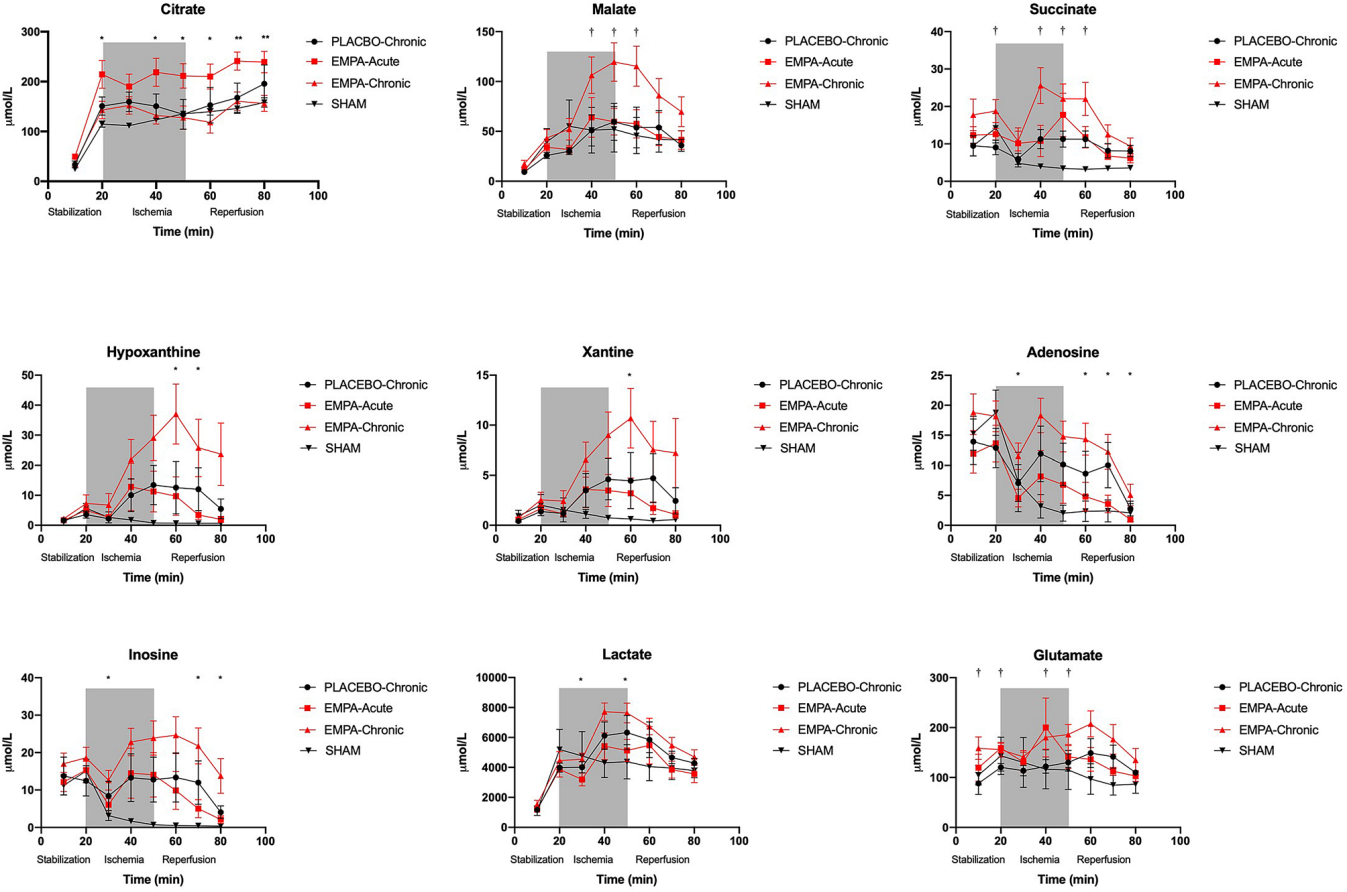
Myocardial interstitial concentrations of metabolites. The myocardial interstitial concentration of TCA cycle intermediates (citrate, succinate and malate) and purine metabolites (adenosine, hypoxanthine, inosine and xanthine) during stabilization, ischemia and reperfusion. Data are mean ± SEM. Statistical significance is shown as * p < 0.05 EMPA-Acute vs. EMPA-Chronic, † p < 0.05 EMPA-Chronic vs. PLACEBO-Chronic.

At the beginning of stabilization, the myocardial interstitial concentration of the tricarboxylic acid (TCA) cycle intermediates citrate, succinate and malate were similar in all groups (Fig. 5), but the concentration of glutamate was significantly higher in EMPA-Chronic compared to placebo and remained so during stabilization. EMPA-Acute increased the myocardial citrate level and EMPA-Chronic increased the succinate level before the induction of ischemia compared to EMPA-Chronic and placebo respectively. During ischemia, the interstitial citrate concentration was significantly higher in the EMPA-Acute group compared to EMPA-Chronic and remained continuously increased during reperfusion. The interstitial concentrations of succinate, malate and glutamate were significantly increased by EMPA-Chronic compared to placebo during ischemia and also in early reperfusion.

At the end of stabilization, the interstitial concentrations of purine metabolites were similar in all study groups. Concentrations of adenosine and inosine were significantly elevated in early ischemia compared with EMPA-Acute. During early reperfusion, the interstitial concentrations of adenosine, inosine, hypoxanthine and xanthine were significantly increased in the EMPA-Chronic group compared to EMPA-Acute.

The interstitial concentration of lactate was significantly elevated during ischemia in EMPA-Chronic compared to EMPA-Acute but not compared to placebo.

**Figure 6:**
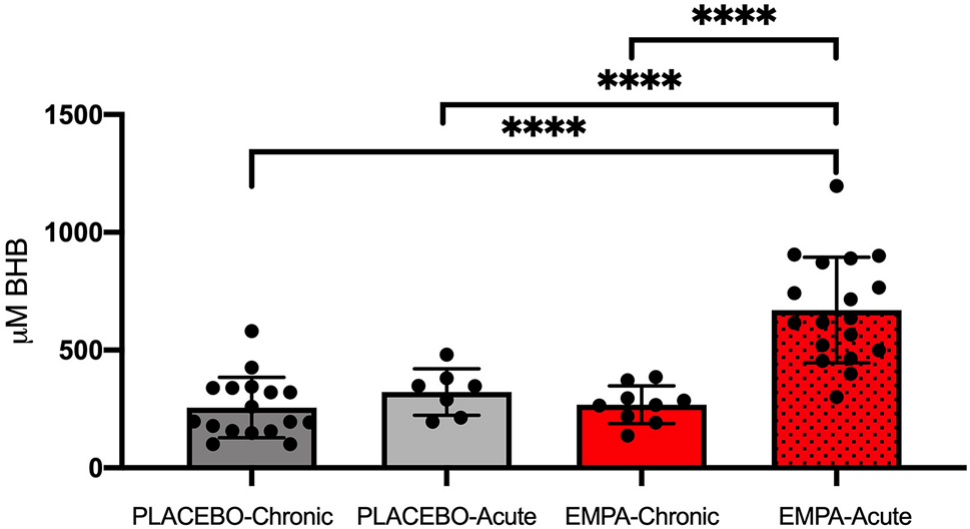
Plasma levels of β-hydroxybutyrate in early reperfusion. β-hydroxybutyrate levels measured from a venous blood sample after 2 h of reperfusion in. PLACEBO (n = 17), PLACEBO-Acute (n = 1.5), EMPA-Chronic (n = 18), EMPA1.5 (n = 9). BHB: β-hydroxybutyrate. Data are mean ± SD. Statistical significance is shown as **** p < 0.0001.

EMPA increased circulating β-OHB acid levels after 1.5 h of its administration. After 7 days of its administration, the level of β-OHB acid was normalized (Fig. 6).

**Figure 7:**
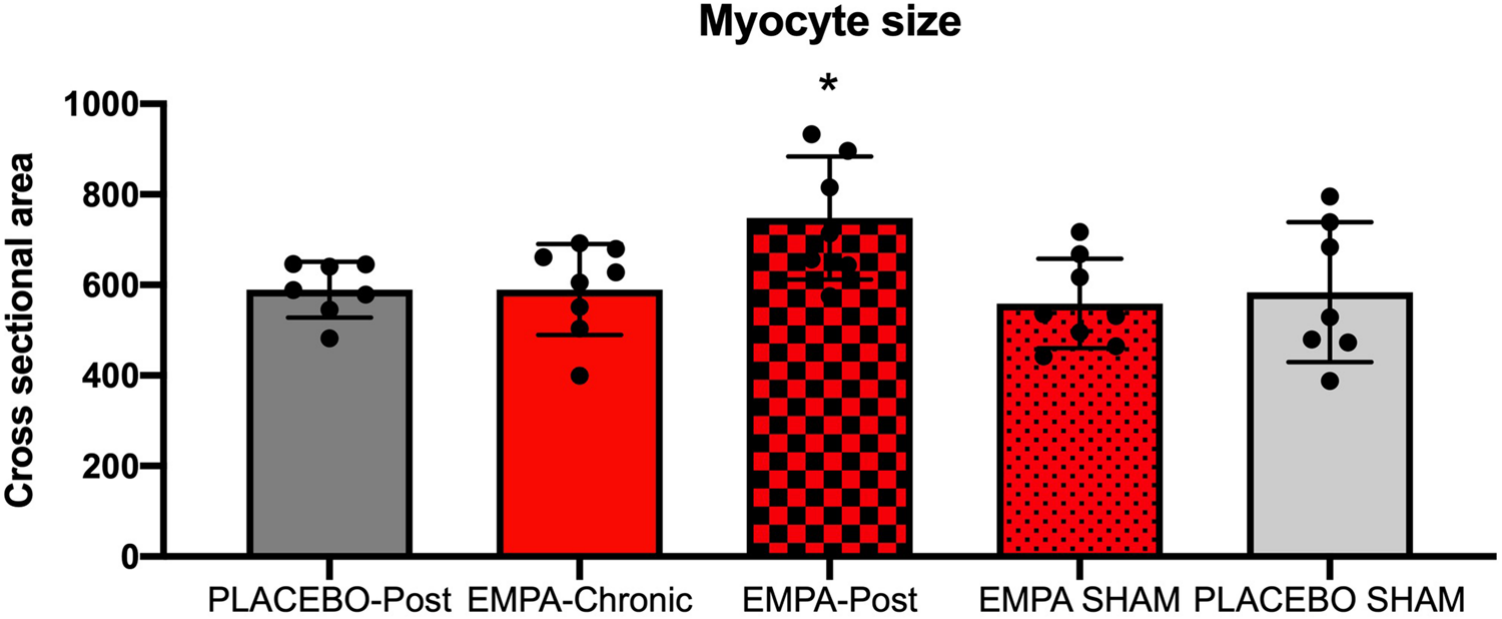
Myocyte cross sectional area. Histological analysis of Myocyte cross sectional area. PLACEBO-Post (n = 7), EMPA-Chronic (n = 7–8), EMPA-Post (n = 7), PLACEBO SHAM (n = 6–7) and EMPA SHAM (n = 8). Mean ± SD. Statistical significance is shown as * p < 0.05, **p < 0.01 vs. PLACEBO-Post.

At 28 days after MI, myocyte cross sectional area was slightly increased in the EMPA-Post group compared to placebo but otherwise similar across the study groups (ANOVA, p < 0.05) (Fig. 7).

## Discussion

The present study demonstrates that continuous treatment with EMPA for 7 days prior to MI reduces IS in an in vivo rat heart model, while a single administration 1.5 h before MI does not. IS reduction is associated with a subsequent improvement of LV function at 28 days post MI. The most notable finding is that administration of EMPA in a post MI period of 28 days improves LV function regardless of time of therapy initiation and hence independently of IS reduction in the in vivo rat heart. The hemodynamic effect of SGLT2 inhibition was associated with an improvement in mitochondrial respiration that was documented in healthy, sham-operated rats. The cardioprotective effect and the effect on mitochondrial respiration seem to be independent of circulating β-OHB levels. Our results confirm that EMPA yields no cardioprotection in the isolated heart. EMPA did not affect mortality rate (Fig. S1).

The mechanisms underlying the cardioprotective effect of treatment with EMPA is of importance in the light of the recent demonstration of a beneficial cardiovascular effect of SGLT2 inhibitors. Notably, the beneficial effects were obtained not only by patients with^8,22^ but also in patients without diabetes^8,9^.

In accordance with previous studies, we found that EMPA administered for one week prior to MI reduced IS in non-diabetic rat hearts^23–25^. Administration of EMPA as a single dose shortly before MI did not offer the same effect^23^. Even though acute intravenous administration of dapagliflozin and canagliflozin is protective^26,27^, this observation may indicate that the main mechanism of EMPA does not work by a primary and direct modulation of the myocardium. This assumption is supported by the fact that administration of SGLT2 inhibitors directly into the perfusate in an ex vivo, Langendorff setting leaves cardioprotection unobtainable^10,12^. We confirmed these findings in a Langendorff setting that eliminated any effect of circulating substances (e.g. metabolic substrates such as β-OHB), since the initial washout with high coronary flow rates rapidly dilutes substances, which are carried over from the body. Hence, the mediating cardioprotective signal appears to be dependent on a whole-body system.

EMPA improves contractility in hypoxic cardiomyocytes^25^. We extended these findings by demonstrating that EMPA enhanced EF irrespective of the time of treatment onset in rats with post infarction compromised LV function. Our findings demonstrated that IS reduction alone is not responsible for the effect. EMPA improved end systolic volume, while end diastolic volume remained unchanged, reflecting increased contractility. In accordance with our findings, canagliflozin increased stroke volume and myocardial efficiency without altering myocardial substrate utilization, i.e. uptake of glucose, fatty acids or ketones, in otherwise healthy swine subjected to IR injury^28^ and alleviated post-ischemic systolic and diastolic function in non-diabetic male rats^27^. Together, these findings suggest that SGLT2 inhibitors have an inherent beneficial modulating class effect that reduces the cardiac derangements following MI.

Expectedly, the mitochondrial complex I and complex I + II linked oxidative phosphorylation (OXPHOS) capacity was impaired by IR injury during early reperfusion after MI. EMPA-Chronic significantly improved mitochondrial function. EMPA-Chronic also improved mitochondrial fatty acid oxidation. The main electron entry sites in the ETS after β-oxidization of fatty acids are complexes I and II via NADH and electron transfer flavoprotein via FADH_2_^29^. Hence, the increased respiration by EMPA might reflect an overall improvement in the electron flow through complex I and II, as reflected in the respiratory coupling efficiency (RCR) and driven by an increase in state 3 respiration. Reduction of the oxidative capacity of fatty acids plays an important role in the development of HF after IR injury^30,31^. This reduction provides an early indication of deranged cardiac mitochondrial performance in HF^30^. EMPA-Chronic improved both complex I and fatty acid respiration.

Because we measured mitochondrial respiration under unloaded resting conditions in the absence of EMPA or its potential mediator, the preserved mitochondrial function may favour a mechanism underlying the improved cardiac function rather than a consequence and that the modulation has already happened at the organ level in the intact body. Supporting this, we observed a significantly improved mitochondrial respiratory capacity in sham animals treated with EMPA compared with sham animals treated with placebo. Thus, our findings seem to represent a specific effect of EMPA on mitochondrial respiratory capacity. We have previously demonstrated a similar effect in an in vitro model^12^.

ATP degradation end products (i.e. adenosine, inosine, hypoxanthine and xanthine) were elevated during ischemia^19^. In accordance with an increased ATP-turnover from the elevated respiratory capacity, in particular during reperfusion when the respiratory chain is recovering^32^, we found an amplified increase in the ATP degradation products in the EMPA-Chronic group. The myocardial interstitial concentrations of TCA cycle intermediates succinate, malate and glutamate were significantly elevated in the EMPA-Chronic compared to placebo during ischemia.

In our experimental rat model, we found that levels of β-OHB increased only transiently after administration of EMPA. Conversely, in the EMPA-Chronic group, circulating β-OHB levels were normalized at the time of exposure to IR with no apparent association with the observed IS reduction. Since we were unable to associate elevated β-OHB levels in plasma with IS reduction and increased mitochondrial respiration, it seems unlikely that the cardioprotective effect should be coupled to increased β-OHB metabolism.

Some limitation must be acknowledged. We used healthy young male animals without comorbidities and without previous exposure to pharmacological treatment. Furthermore, we used a supra-therapeutic dose of EMPA to investigate the cardioprotective properties and mitochondrial modulatory effects on MI and post-MI HF. During several of the study procedures, the animals underwent anesthesia with volatile anaesthetics, which has been shown to be cardioprotective^33^. However, we found no differences in length of anesthesia or doses of sevoflurane between our study groups.

We found no differences in the number of mitochondria, measured by citrate synthase activity. A biomarker such as citrate synthase may not be an optimal marker for mitochondrial content across varying pathological conditions such as IR^34^. However, the same constraint relates to all markers that seem to have similar validity as citrate synthase^35^. To circumvent the limitation, we calculated the respiratory control ratio, as a reliable measure of respiratory coupling efficiency, independently from the number of mitochondria. Mitochondrial respiration was measured 30 min after reperfusion, whereas infarct size was evaluated after 2 h. The damaging process of reperfusion may extend beyond 30 min and thus mitochondrial respiration may still be significant as part of the cardioprotective mechanism, as shown in the chronic experimental series. However, the time discrepancy may explain that we found no IS reduction in EMPA-Acute animals, while mitochondrial respiratory capacity was similar to that observed in the EMPA-Chronic group. Similarly, it might explain that concentrations in intermediary metabolites were different in EMPA-Acute and EMPA-Chronic, while we found no difference in mitochondrial respiration.

The microdialysis samples were collected in 10-min spans to ensure sufficient material for analysis. This may limit the interpretation as a result of low temporal resolution compared to the rapid changes that occur during early reperfusion. Catheter implantation and surgical procedures may per se affect levels of myocardial interstitial metabolites^36^ and may challenge the interpretation of the physiology in our specific experimental setup. The influence was observed initially and was similar in all study groups, whereas no increase was observed in the sham group during ischemia. Hence, we considered the differences between the study groups during ischemia and reperfusion valid. Furthermore, microdialysis is not the most accurate method to estimate exact concentrations in tissues, but we chose the continuous measurement approach from the same animal to assess dynamic changes.

We did not investigate causes of mortality during the experiments, specifically we did not monitor arrhythmicity, which might have provided information about differences in causes of mortality between groups.

## Conclusion

EMPA yields cardioprotection against acute IR injury in vivo but not ex vivo, indicating dependency of an intact body and an indirect effect by a delayed intrinsic cardioprotective mechanism. The protection persists in the failing rat heart by restoring systolic function and the effect is present irrespective of infarct size reduction. The cardioprotective effect is associated with enhanced mitochondrial respiratory capacity. Our data support a beneficial effect of EMPA in non-diabetic individuals with post infarction left ventricular dysfunction.

## Supplementary Information

Supplementary Information

## Data Availability

All data included in this article is displayed or can be displayed upon request.

## Abbreviations

AAR: Area at risk
ADP: Adenosine diphosphate
ATP: Adenosine triphosphate
ANOVA: Analysis of variance
β-OHB: β-Hydroxybutyrate
CS: Citrate synthase
EF: Ejection fraction
ETS: Electron transport system
HF: Heart failure
IR: Ischemia reperfusion
IS: Infarct size
LAD: Left anterior descending coronary artery
LV: Left ventricle
MI: Myocardial infarction
mPTP: Mitochondrial permeability transition pore
RCR: Respiratory control ratio
SD: Standard deviation
SGLT2: Sodium glucose Co-Transporter 2
TTC: 2,3,5-Triphenyltetrazoliumchloride
TCA: Tricarboxylic acid cycle
Vol_d_: End diastolic volume
Vol_s_: End systolic volume
