# Supplementary material for: Cardioprotective effects of empagliflozin after ischemia and reperfusion in rats
Source: Sci Rep. 2021 May 5;11:9544. doi: 10.1038/s41598-021-89149-9 (PMC8100147; doi:10.1038/s41598-021-89149-9)
Supplement: Supplementary file 1 — Supplementary Information [file 41598_2021_89149_MOESM1_ESM.docx]

Cardioprotective effects of Empagliflozin after

Ischemia and Reperfusion in rats.

Supplementary Material

**Short Title:**

Cardioprotective effects of Empagliflozin

**Authors:**

*Jacob Marthinsen Seefeldt, MS, PhD student^1,2^ (<https://orcid.org/0000-0002-0633-5878>)

Thomas Ravn Lassen, M.D. PhD^1^ (<https://orcid.org/0000-0003-4765-0601>)

Marie Vognstoft Hjortbak, M.D. PhD student^1,2^ (<https://orcid.org/0000-0002-3434-1394>)

Nichlas Riise Jespersen, M.D. PhD^1^ (<https://orcid.org/0000-0003-2502-2102>)

Frederikke Kvist, MS ^1,2^ (<https://orcid.org/0000-0002-6874-8547>)

Jakob Hansen, M.Sc. PhD^3,2^ (https://orcid.org/0000-0002-4115-2858)

Hans Erik Bøtker, Prof., M.D., PhD^1,2^ (<https://orcid.org/0000-0001-6358-8962>)

Corresponding author:

Jacob Marthinsen Seefeldt

Telephone: +45 41 17 86 33

Email: [Jacob.Seefeldt@clin.au.dk](mailto:Jacob.Seefeldt@clin.au.dk)

Address: Thunøgade 30, st. tv, 8000 Aarhus C, Denmark

## Supplemental figures

**Figures:**

**Figure S1: A CONSORT style diagram of animals included in the different experimental series.**

Reasons for exclusion of animals are shown in all groups.

a) In vivo infarct size series. 66 animals were started into the study, of which 51 completed. Chi-square test on mortality in EMPA vs. PLACEBO, p=0.57. b) Post-MI compromised LV function series. 50 animals were started into the study, of which 35 completed. Chi-square test on mortality in EMPA vs. PLACEBO, p=0.43. c) Mitochondrial respiratory capacity and microdialysis series. 50 animals were started into the study, of which 29 completed. Chi-square test on mortality in EMPA vs. PLACEBO, p=0.70.

EMPA7: Empagliflozin (30mg/kg/day) administered for 7 days prior to ischemia. EMPA1.5: Empagliflozin (30mg/kg) administered as a single bolus 1.5 hours prior to ischemia. EMPA POST: Empagliflozin (30mg/kg) administered as a single bolus after reperfusion in a) and during 28 days follow up in b). EMPA SHAM: Empagliflozin 30mg/kg/day) administered after sham surgery.

PLACEBO7: Vehicle administered for 7 days prior to ischemia. PLACEBO1.5: Vehicle administered as a single bolus 1.5 hours prior to ischemia. PLACEBO POST: Vehicle administered as a single bolus after reperfusion in a) and during 28 days follow up in b). PLACEBO SHAM: Vehicle administered after sham surgery. HRR: High Resolution Respirometry. Reason for HRR exclusion was no HRR signal.

**a**


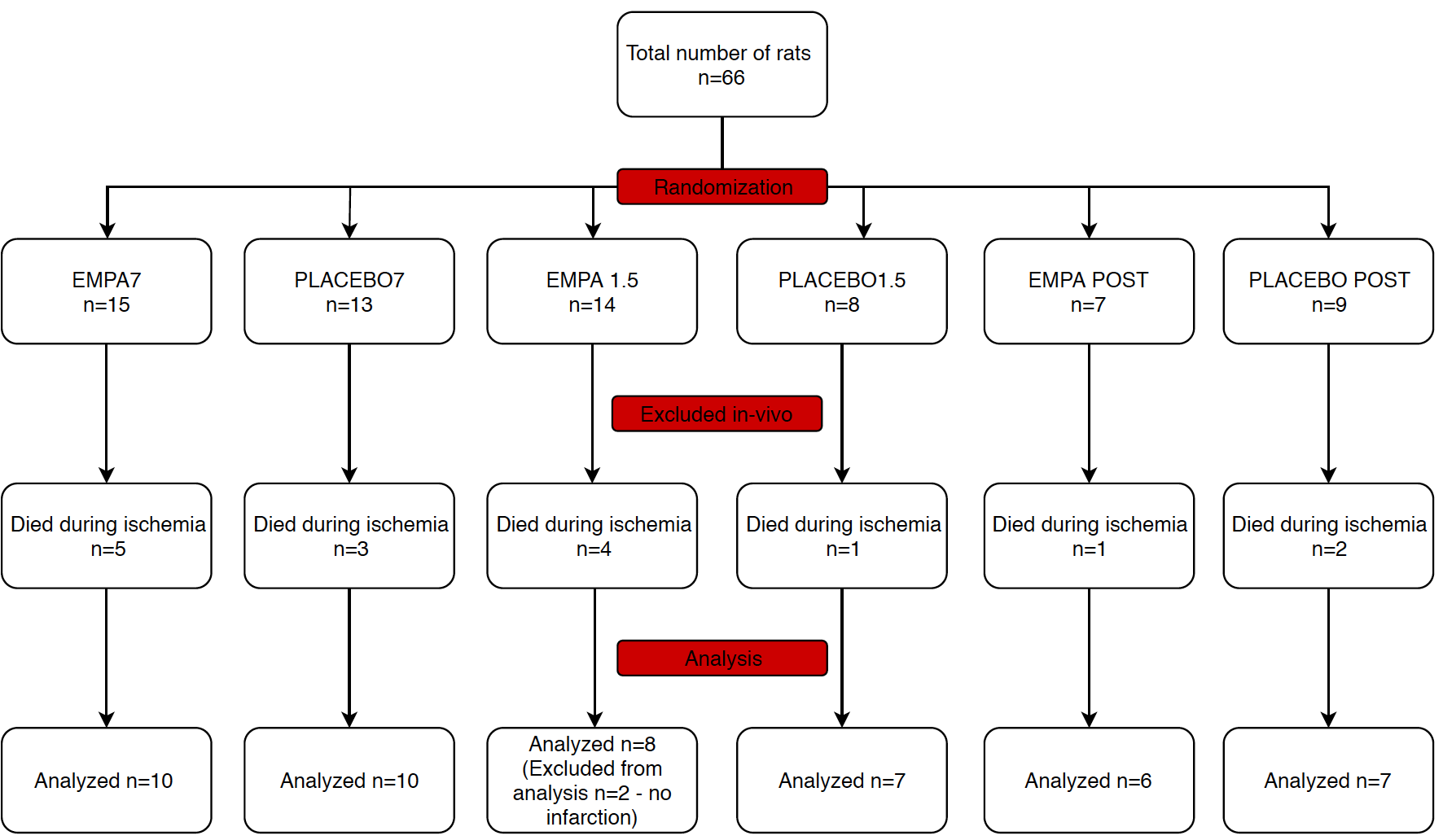


PLACEBO-Post

n=9

EMPA-Post

n=7

PLACEBO-Acute

n=8

EMPA-Acute

n=14

PLACEBO-Chronic

n=13

EMPA-Chronic

n=15

B)

**b**

**a**


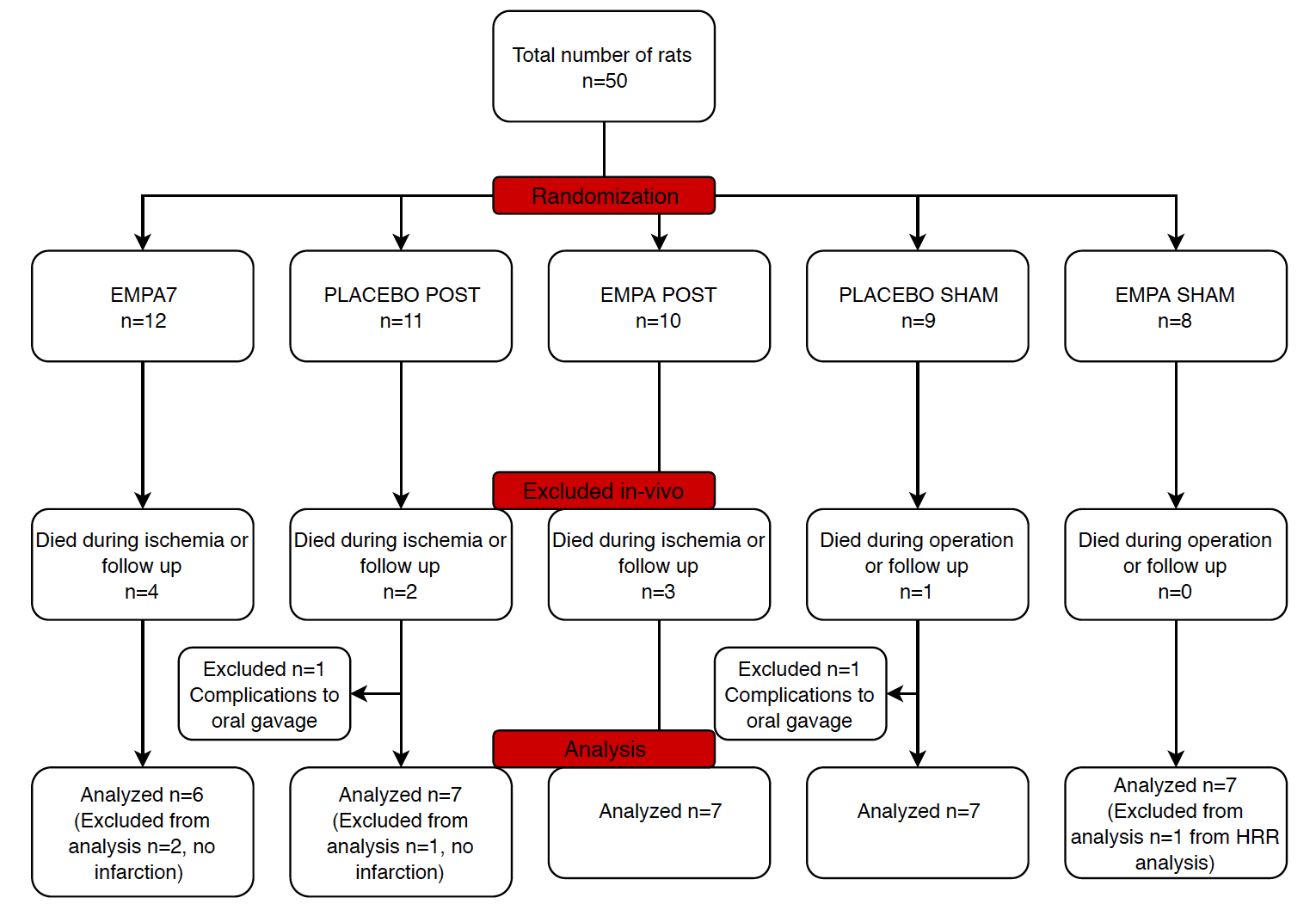


PLACEBO-Post

n=11

EMPA-Post

n=10

EMPA-Chronic

n=12

28 days

28 days

28 days

28 days

28 days

C)

**c**


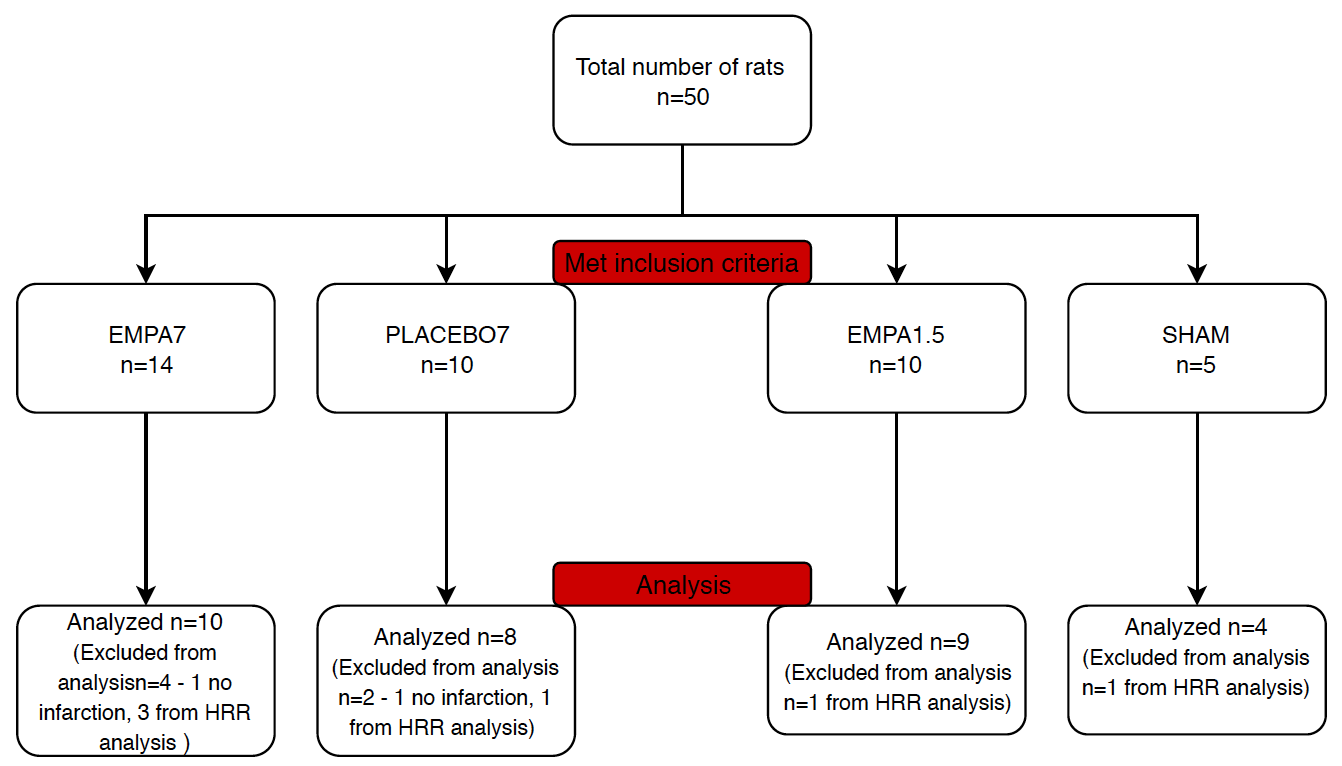


EMPA-Chronic

n=14

PLACEBO-Chronic

n=10

EMPA-Acute

n=10

**Figure S2: Pilot myocardial infarct size following ischemia reperfusion *in vivo***

Histological evaluation of infarct size with 2,3,5-triphenyltetrazolium chloride (TTC) staining, 2 hours after reperfusion in PLACEBO-Chronic (n=6); EMPA-Chronic (n=4) after 7 days repeated administration. AAR area at risk; IS infarct size. Mean ± SD. Statistical significance is shown as * p < 0.05, ** p < 0.01, **** p < 0.0001.

**Figure S3: Myocardial infarct size and hemodynamic assessment following ischemia reperfusion ex vivo.**

a) Histological evaluation of infarct size with 2,3,5-triphenyltetrazolium chloride (TTC) staining, 2 hours after reperfusion in PLACEBO-Chronic (n=9) and EMPA-Chronic (n=9). AAR area at risk; IS infarct size.

b) In line assessment of hemodynamic function. LVDP left ventricular developed pressure; RPP rate pressure product; dPdt rate of pressure change during isovolumetric contraction.

Mean ± SD. Statistical significance is shown as as * p < 0.05, ** p < 0.01, **** p < 0.0001.

**a**

**b**
